# Glymphatic System Dysfunction in Thyroid‐Associated Ophthalmopathy: A Multimodal MRI Study

**DOI:** 10.1111/cns.70650

**Published:** 2025-11-09

**Authors:** Lijie Zhang, Jiaqi Yao, Yiming Qi, Yongheng Luo, Jun Liu

**Affiliations:** ^1^ Department of Radiology The Second Xiangya Hospital of Central South University Changsha China; ^2^ Department of Radiology The Second Affiliated Hospital of Xinjiang Medical University Xinjiang China; ^3^ Clinical Research Center for Medical Imaging in Hunan Province Changsha China; ^4^ Department of Radiology Quality Control Center in Hunan Province Changsha China

**Keywords:** choroid plexus, diffusion tensor imaging along the perivascular space, glymphatic system, thyroid‐associated ophthalmopathy

## Abstract

**Objective:**

To investigate glymphatic system (GS) alterations in thyroid‐associated ophthalmopathy (TAO) patients and their links to clinical biomarkers, with the aim of understanding underlying mechanisms and potential therapeutic strategies.

**Methods:**

This study included 28 active patients (AP), 25 inactive patients (IP) with TAO, and 37 healthy controls (HC), matched for age, sex, and educational level. Glymphatic function was evaluated using choroid plexus volume (CPV), diffusion tensor imaging along the perivascular space (DTI‐ALPS) index, and coupling between global blood‐oxygen‐level‐dependent signals and cerebrospinal fluid signals (gBOLD‐CSF coupling). General linear regression analysis was conducted to explore the relationships between the GS and clinical parameters.

**Results:**

Significant differences were found in the ALPS index between the patient groups and the HC. Both the left (ALPS_L) and right (ALPS_R) ALPS indices, as well as the average ALPS index, were significantly lower in the AP and IP groups than in the HC (*p* < 0.01). The overall difference across all three groups was also significant (*p* < 0.01). However, no significant differences were observed between the AP and IP groups. However, no significant differences in CPV were observed between either the AP or IP group and the HC. Both the AP and IP groups exhibited lower gBOLD‐CSF coupling than the HC (*p* = 0.001; *p* = 0.013). Significant correlations existed between GS function and clinical parameters in both groups.

**Conclusion:**

TAO patients demonstrate significant GS impairments linked to neuropsychological symptoms and sleep disturbances. Targeting GS function may improve quality of life in TAO patients.

## Graphical abstract

TAO patients exhibit impaired glymphatic function, with reduced DTI‐ALPS indices and gBOLD‐CSF coupling, but unchanged choroid plexus volume. These alterations correlate with neuropsychological symptoms and sleep disturbances. Restoring glymphatic activity may offer a therapeutic approach to enhance patient outcomes.

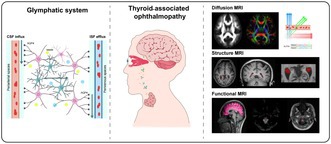

## 1 Introduction

Thyroid‐associated ophthalmopathy (TAO), an autoimmune inflammatory orbital disease closely linked to thyroid dysfunction, primarily affects intraorbital muscles, adipose tissue, and lacrimal glands [1]. Epidemiological studies have demonstrated the high prevalence of TAO in Graves' disease (GD) populations across both Asian and European demographics, ranking it as the most prevalent orbital disorder in adults [2, 3].

The clinical spectrum of TAO extends beyond characteristic ocular manifestations such as eyelid swelling and retraction to encompass neuropsychological abnormalities, including depression, anxiety, and memory impairment [4]. Recent advancements in multimodal magnetic resonance imaging (MRI) have facilitated investigations into potential cerebral structural and functional alterations in TAO patients. Current functional MRI (fMRI) studies have revealed abnormalities in regional neural activity and disrupted functional integration within specific brain regions of TAO patients [5, 6]. Furthermore, structural and diffusion MRI techniques have uncovered microstructural alterations in neural pathways associated with vision, including changes within the visual processing, executive control, dorsal attention, and sensorimotor networks [6, 7, 8]. These findings suggest that TAO may extend beyond orbital manifestations to exert systemic effects on the nervous system, particularly involving cerebral structures.

Emerging research in recent years has identified a specialized fluid exchange and transport network in the brain, termed the glymphatic system (GS) [9]. This system facilitates cerebrospinal fluid (CSF) influx into brain parenchyma via periarterial spaces, subsequently clearing metabolic waste through interstitial fluid drainage to meningeal glymphatic vessels and deep cervical lymph nodes [10]. Growing evidence reveals intricate bidirectional connections between ocular pathologies and cerebral alterations [11]. Pioneering studies have demonstrated that the posterior eye compartment possesses a unique GS directly interconnected with the cerebral meningeal GS network [12], establishing an immunological conduit between the eye and brain. This breakthrough not only fills critical gaps in ocular immunology but also provides novel therapeutic perspectives for both ophthalmic and central nervous system (CNS) disorders. Recent investigations further reveal GS impairment in glaucoma patients [13], yet systematic exploration of GS functional changes in TAO remains conspicuously scarce.

Advancements in neuroimaging technologies now enable non‐invasive evaluation of GS structure and function. The Diffusion tensor imaging analysis along the perivascular space (DTI‐ALPS) index has emerged as a validated biomarker for CNS assessing waste clearance capacity in the CNS [14, 15, 16]. This technique quantifies diffusion metrics in projection and association fibers perpendicular to paraventricular perivascular spaces, providing reliable functional assessment of the GS [17]. Concurrently, the choroid plexus (CP)—a vascularized epithelial network serving as a blood–brain immune cell gateway—has been identified as a key driver of GS function [18]. Recent discoveries also reveal coupled dynamics between global blood oxygen‐level‐dependent (gBOLD) signals and CSF inflow kinetics, with their synchronization strength serving as a novel indicator of GS activity [19].

Although previous studies have reported abnormalities in the ALPS index in patients with TAO [20], earlier research has focused solely on this single metric. To address this gap, our study is the first to employ three non‐invasive multimodal MRI techniques to indirectly evaluate changes in GS function in TAO patients from structural, functional, and diffusion perspectives. We hypothesize that patients with TAO exhibit GS dysfunction, which is associated with neuropsychological symptoms and sleep disturbances. Through these assessments, we hope to provide new theoretical insights into the clinical diagnosis and treatment of TAO, particularly in understanding how the condition affects the brain and nervous system beyond the ocular symptoms, thus offering valuable references for the development of future treatment strategies.

## 2 Materials and Methods

### 2.1 Participants

This study was approved by the Ethics Review Committee of the Second Xiangya Hospital of Central South University and all participants provided written informed consent in accordance with the Declaration of Helsinki. Patients with TAO and healthy controls (HC) were enrolled from March 2024 to January 2025. The enrollment criteria are as follows: (1) Patients clinically diagnosed with TAO based on the clinical guidelines of the European Group on Graves' Orbitopathy (EUGOGO); (2) Availability of complete clinical data for Clinical Activity Score (CAS) grading; (3) Absence of contraindications for MRI examination; (4) Ability to complete all neuropsychological scale tests and the entire MRI scanning process; (5) Right‐handedness; (6) No history of drug or alcohol abuse. The exclusion criteria are as follows: (1) Signs and medical history of other eye diseases, such as glaucoma and cataracts; (2) history of ophthalmic surgery or trauma; (3) high hyperopia (> +3D); high myopia (< −5D); (4) history of psychiatric or neurological disorders, such as depression and bipolar disorder; (5) brain anatomical abnormalities, such as tumors; (6) contraindications for MRI scans; (7) poor image quality; (8) incomplete clinical data.

All participants provided written informed consent and were confirmed to be right‐handed. Demographic and clinical data, including age, sex, disease duration, educational attainment, and thyroid function laboratory results, were systematically collected. Clinical symptoms were assessed in the patient group using standardized clinical rating scales and self‐report questionnaires, including the Hamilton Anxiety Rating Scale (HAMA), Hamilton Depression Rating Scale (HAMD), and Pittsburgh Sleep Quality Index (PSQI). Additionally, TAO patients were also evaluated using a quality of life (QoL) questionnaire, which included visual function (VF) and appearance (AP) questions.

Age, sex, and educational attainment‐matched HC were recruited from the community. Disease duration was calculated from the initial onset of ocular symptoms. TAO activity was assessed through the 7‐point clinical CAS combined with orbital MRI, consistent with clinical guidelines [21]. In this study, the research cohort was divided into the active TAO group (AP), the inactive TAO group (IP), and the HC group, which more accurately reflects the clinical characteristics of the disease and yields more precise results [22]. The flowchart summarizing the patient enrollment process is shown in Figure S1.

### 2.2 Imaging Acquisition

High‐resolution MRI data were acquired using a Siemens Trio 3 T scanner (imaging parameters summarized in Table S1). To mitigate confounding physiological effects on GS function, all participants were instructed to maintain a standardized supine position and practice relaxed breathing throughout the scan protocol, minimizing artifacts from respiratory variations, and postural changes—factors previously shown to modulate GS fluid dynamics in preclinical models [23].

### 2.3 DTI‐ALPS

The calculations were performed following the methodology from previous studies [24]. The DTI data in DICOM format were first converted to NIFTI format. Using an in‐house bash script incorporating tools from FSL (version 6.0.4; https://fsl.fmrib.ox.ac.uk/fsl/fslwiki/) and MRtrix3, we performed a series of preprocessing steps, such as eddy current correction, motion correction, and skull stripping. Next, the fractional anisotropy (FA) maps and the *x*‐, *y*‐, and *z*‐axis diffusivity distribution plot were generated by using the FSL command line tool “dtifit.” After each subject's FA maps were registered with the JHU‐ICBMFA template, the transformation was applied to all diffusivity profiles utilizing the FSL command line tool “flirt.” Spherical regions of interest (ROI) with a diameter of 5 mm were automatically placed in the aligned diffusivity maps for all subjects. Diffusivity values along the *x*, *y*, and *z* directions within these ROI were extracted to compute the ALPS index.

### 2.4 CP Volume Calculation

The CP is the primary site for CSF secretion and is considered an indirect imaging biomarker for evaluating CSF production and toxic clearance [25]. We used FreeSurfer version 7.3.2 to automatically estimate the structural volumes (http://surfer.nmr.mgh.harvard.edu/). The preprocessing steps followed the standard template within the subject, including steps such as skull stripping, Talairach transformation, and the creation of spherical mappings. To reduce inter‐subject variability, the choroid plexus volume (CPV) was normalized by computing the ratio of CPV to intracranial volume (ICV); the CP ratio was calculated using the following formula: CP ratio = (CPV/ICV) × 1000 [26]. This normalized measure is referred to as the CP ratio throughout the manuscript.

#### 2.4.1 gBOLD Data Preprocessing

Resting‐state fMRI (rs‐fMRI) and 3D‐T1‐weighted imaging (3D‐T1WI) data underwent standardized preprocessing using Statistical Parametric Mapping (SPM12; www.fil.ion.ucl.ac.uk/spm) and the Data Processing and Analysis for Brain Imaging toolbox (DPABI v6.0; http://rfmri.org/dpabi) [27, 28].

#### 2.4.2 Definition of ROI

ROIs for cortical gray matter were defined based on the Harvard‐Oxford cortical structural atlas [29]. T1‐weighted images were linearly registered to the rs‐fMRI data to generate cortical region masks, which were then non‐linearly co‐registered to Montreal Neurological Institute (MNI) space using SPM12. The resulting matrices were saved for further analysis. The ROIs in MNI space were then transformed back to the original rs‐fMRI space. Previous studies have confirmed that this signal reflects CSF flow and is highly sensitive at the bottom of the cerebellum [30]. Therefore, we selected the bottom slices of the fMRI images to capture the signal. Two radiologists, blinded to the clinical data, manually defined the CSF ROI based on the rs‐fMRI images and performed intraclass correlation coefficient (ICC) analysis (Figure S2).

#### 2.4.3 Quantification of gBOLD–CSF Coupling Strength

After extracting the rs‐fMRI signals from cortical gray matter and CSF, we computed the cross‐correlation function between gBOLD and CSF signals using. Pearson correlation to assess their coupling strength within a specific time lag range (−10 s to +10 s). Since the negative peak at +4 s and the positive peak at −4 s exhibit equal amplitude, we focused on the gBOLD‐CSF correlation at the negative peak to quantify the coupling strength. In line with previous studies [31], we also evaluated the cross‐correlation function between the negative derivative of the gBOLD signal and the CSF signal. To determine the statistical significance of the gBOLD‐CSF correlation, we applied a permutation strategy by randomly pairing gBOLD and CSF signals from different subjects. This procedure was repeated 10,000 times to generate a null distribution based on these correlations, with all calculations carried out using MATLAB (version R2022a). Subsequently, the gBOLD‐CSF coupling strength values were calculated individually.

### 2.5 Statistical Analyses

Statistical analyses were performed using IBM SPSS Statistics (version 26.0). Group comparisons among AP, IP, and HC were conducted using one‐way ANOVA for normally distributed continuous variables (with adjustment for covariates such as age, sex, and education level), followed by Bonferroni‐corrected pairwise post hoc tests for multiple comparisons. The Kruskal–Wallis test was applied for non‐normally distributed data. Categorical variables were assessed using the chi‐square test. For comparisons between two groups, independent samples *t*‐tests were used for normally distributed continuous variables, and the Mann–Whitney *U* test was used for non‐normally distributed variables.

Interrater reliability of gBOLD–CSF coupling measurements was determined via the ICC within regions of interest in the CSF, and mean values were subsequently used in the analyses. Correlations between GS parameters and clinical variables were examined using general linear regression. Statistical significance was defined as a two‐tailed *p*‐value < 0.05.

## 3 Results

### 3.1 Demographic and Clinical Measurements

This study included 53 adults diagnosed with TAO (28 AP and 25 IP) and 37 HC. Demographic analysis revealed no significant differences between the three groups in terms of age (*p* = 0.417), gender distribution (*p* = 0.760), and education level (*p* = 0.598). Significant differences were observed in sleep quality between the HC and both the AP and IP groups (*p* < 0.001 for both). Notably, the AP group had lower scores on the QoL visual scale compared to the IP group, with a significant difference. Detailed information is summarized in Table S2.

### 3.2 Group Differences in ALPS Index

**FIGURE 1:**
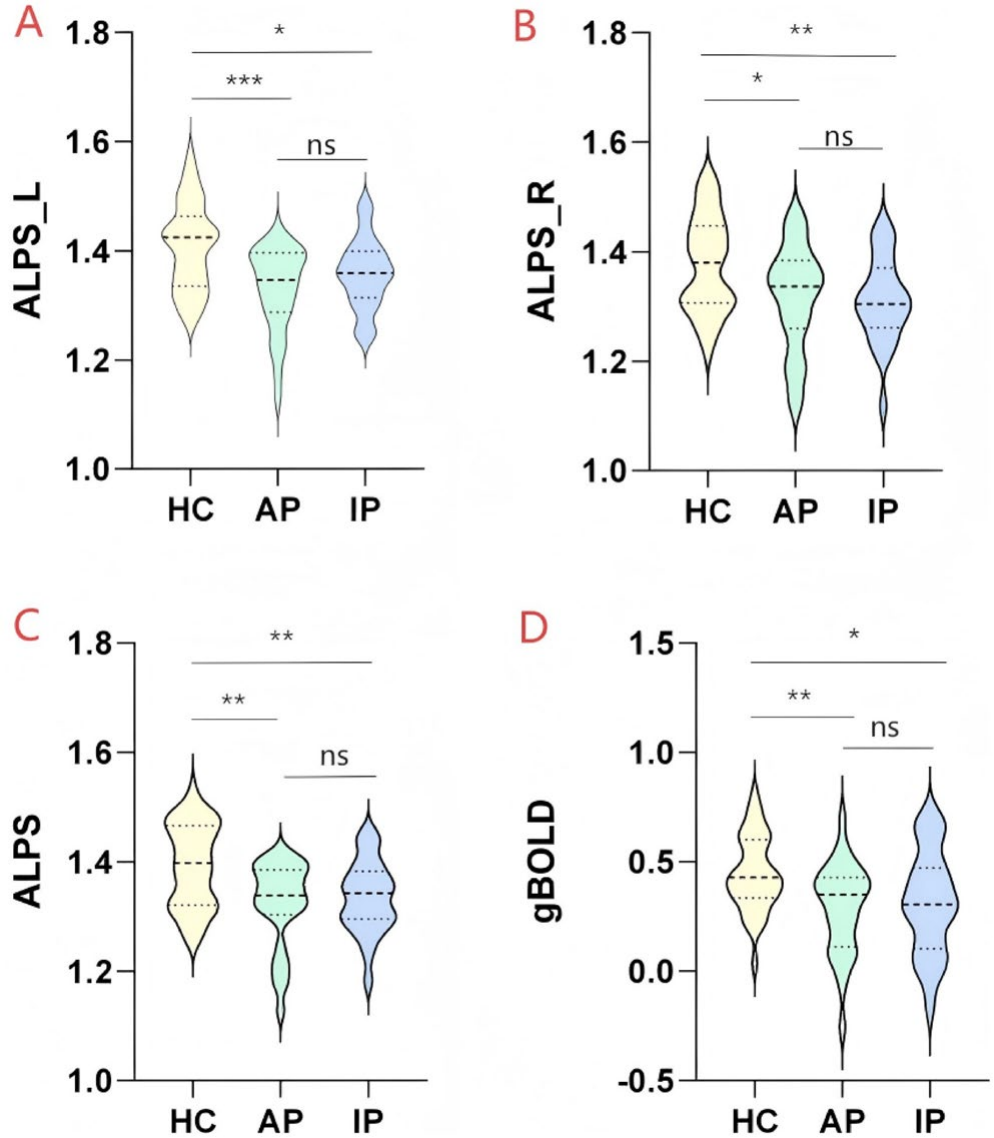
Comparison of GS markers among HC, AP, and IP groups. (A–C) AP and IP groups showed significantly reduced ALPS indices compared to the HC group, but no significant differences were found between patients. (D) AP and IP groups showed significantly reduced gBOLD‐CSF compared to the HC group, but no significant differences were found between patients. ALPS, along the perivascular space; AP, active patients; gBOLD, global blood‐oxygen‐level‐dependent signals; GS, glymphatic system; HC, healthy controls; IP, inactive patients; L, left; R, right. Significance of *, **, *** indicates the statistical significance of *p*‐values when *p < 0.05, **p < 0.01, ***p < 0.001 respectively.

Compared to the HC group, both the AP and IP groups exhibited significantly lower ALPS_L (*p* < 0.001; *p* = 0.009), ALPS_R (*p* = 0.009; *p* = 0.004), and the average ALPS index (*p* = 0.001; *p* = 0.003). However, no significant differences were found between the AP and IP groups in the left, right, and average ALPS indices (Table 1 and Figure 1). Additionally, the ALPS values of the left hemisphere were higher than those of the right hemisphere [32].

**TABLE 1:** Between‐group differences in glymphatic system markers.

Characteristics	AP (*n* = 28)	IP (*n* = 25)	HC (*n* = 37)	*p*			
				AP vs. IP	AP vs. HC	IP vs. HC	AP vs. IP vs. HC
ALPS_L	1.330 ± 0.077	1.357 ± 0.064	1.409 ± 0.079	0.170	< 0.001	0.009	< 0.001
ALPS_R	1.316 ± 0.096	1.312 ± 0.078	1.377 ± 0.088	0.872	0.009	0.004	0.005
ALPS	1.323 ± 0.078	1.335 ± 0.066	1.393 ± 0.077	0.562	0.001	0.003	< 0.001
CPV_L (mm^3^)	524.918 ± 153.678	528.304 ± 180.758	506.251 ± 141.656	0.942	0.614	0.593	0.832
CPV_R (mm^3^)	587.311 ± 143.156	585.144 ± 182.243	571.465 ± 142.246	0.962	0.657	0.740	0.784
CP ratio	0.934 ± 0.189	0.937 ± 0.199	0.923 ± 0.235	0.955	0.836	0.804	0.975
gBOLD	0.288 ± 0.207	0.313 ± 0.235	0.445 ± 0.172	0.678	0.001	0.013	0.004

Abbreviations: ALPS, along the perivascular space; AP, active patients; CP, choroid plexus; CPV, choroid plexus volume; gBOLD, global blood‐oxygen‐level‐dependent signals; HC, healthy controls; IP, inactive patients; L, left; R, right.

### 3.3 Group Differences in CPV

There were no significant differences in ICV between the AP, IP, and HC groups (*p* = 0.699). However, the AP and IP groups showed no significant differences in the three CP parameters (CPV_L, CPV_R, and CP ratio) compared to the HC (*p* = 0.832, *p* = 0.784, and *p* = 0.975) (Table 1). The mean values for all three CP parameters were higher in both patient groups compared to the HC group.

### 3.4 Group Differences in gBOLD‐CSF

**FIGURE 2:**
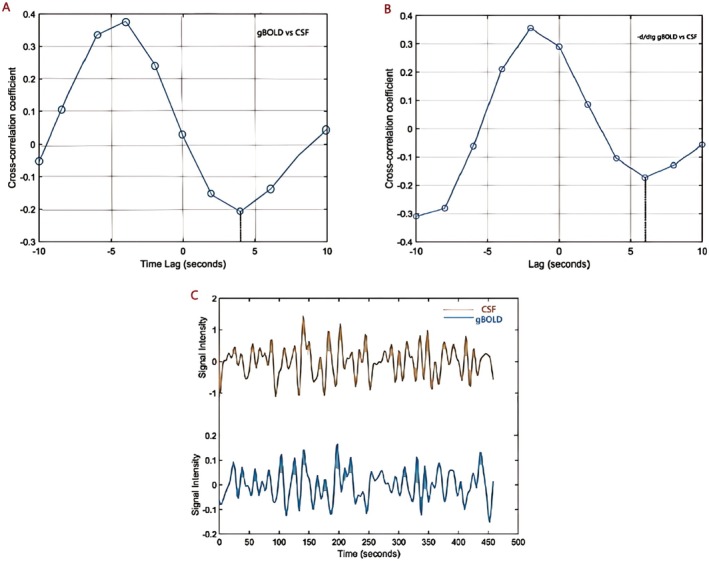
The gBOLD signal is coupled with CSF changes. (C) gBOLD and CSF signals showed coupled amplitude changes, with a significant positive peak at 4s (R = 0.284, *p* < 0.001) and a negative peak at 6s (R = –0.345, *p* < 0.001), confirming their systemic coupling (A–B). CSF, cerebrospinal fluid; gBOLD, global blood‐oxygen‐level‐dependent signals.

To investigate whether the gBOLD signals of all participants were closely associated with variations in the CSF signal, we extracted gBOLD and CSF fMRI signals from both the whole‐brain gray matter and CSF regions. The gBOLD and CSF signals displayed synchronized amplitude fluctuations. The gBOLD and CSF signals exhibited corresponding amplitude changes. The average characteristic showed a positive peak at a 4‐s delay (*R* = 0.284, *p* < 0.001; permutation test: 10,000) (Figure 2A), while a negative peak appeared at a 6‐s delay (*R* = 0.345, *p* < 0.001; permutation test: 10,000, black dashed line) (Figure 2B). The patterns displayed by both correlation functions were consistent with the data, confirming the systemic coupling between global brain signals and CSF flow (Figure 2C), in line with previous studies. The average ICC for gBOLD‐CSF coupling was 0.907, indicating strong agreement between the two researchers. The previous literature used the negative peak correlation value as an indicator of coupling strength for analysis [33]. In this study, the coupling strength indicators of both the AP group and the IP group were lower than those of the HC (*p* = 0.001; *p* = 0.013).

### 3.5 Correlation Between GS Function and Clinical Parameters

**FIGURE 3:**
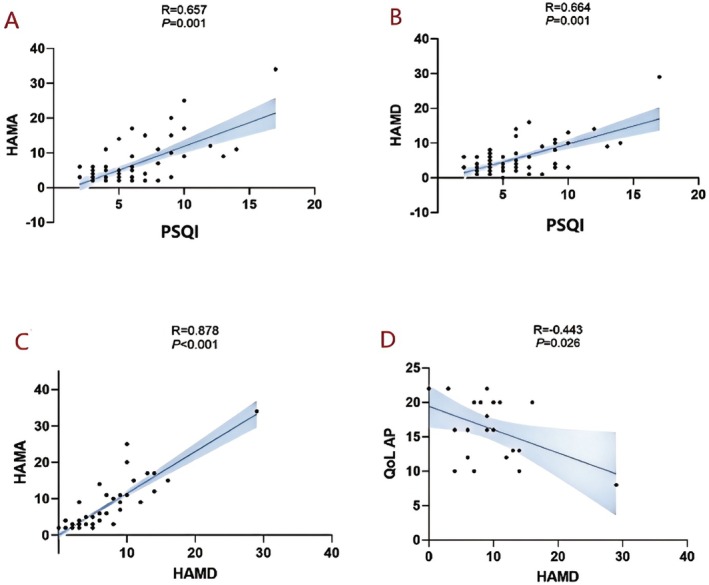
Correlations between various clinical parameters in IP. (A–B) Higher HAMD and HAMA scores in the IP group were also significant positively correlated with higher sleep scores (R = 0.664, *p* = 0.001; R = 0.657, *p* = 0.001). (C) A strong positive correlation was observed between HAMD and HAMA scores (R = 0.878, *p* < 0.001). (D) HAMD scores demonstrated a moderate negative correlation with QoL VF scores (R = −0.443, *p* = 0.026). HAMA, hamilton anxiety rating scale; HAMD, hamilton depression rating scale; IP, inactive patients; PSQI, pittsburgh sleep quality index; QoL AP, quality of life questionnaire of appearance.

**FIGURE 4:**
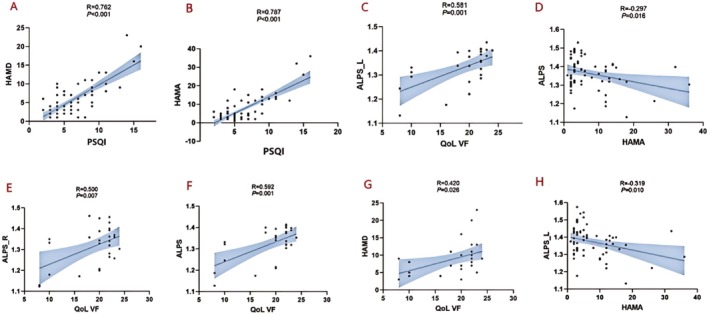
Associations between GS markers and clinical parameters in AP. (A–B) Higher HAMD and HAMA scores in the IP group were also significantly positively correlated with higher sleep scores(R = 0.762, *p* < 0.001; R = 0.787, *p* < 0.001). (C) HAMD scores demonstrated a moderate positive correlation with QoL VF scores (R = 0.581, *p* = 0.001). (D, H) ALPS_L and ALPS were significantly negatively correlated with HAMA (R = –0.319, P=0.010; R = –0.297, *p* = 0.016). (E–G) ALPS_L, ALPS_R, and the ALPS index were positively correlated with GO‐QoL VF (R = 0.561, *p* = 0.001; R = 0.500, *p* = 0.007; R = 0.592, *p* = 0.001). AP, active patients; ALPS, along the perivascular space; HAMA, hamilton anxiety rating scale; HAMD, hamilton depression rating scale; L, left; PSQI, pittsburgh sleep quality index; QoL VF, quality of life questionnaire, visual function; R, right.

**FIGURE 5:**
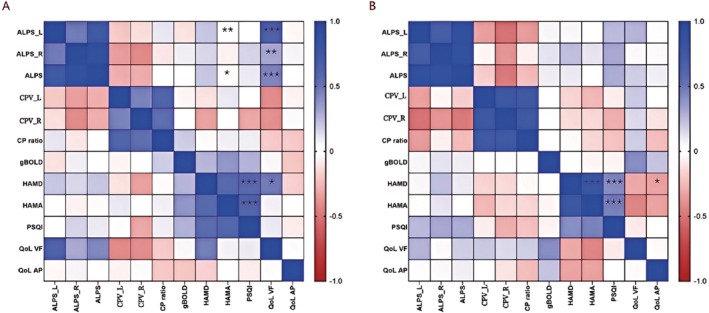
The correlation analysis results between all GS markers and clinical parameters in AP (A) and IP (B). ALPS, along the perivascular space; AP, active patients; CP, choroid plexus; CPV, choroid plexus volume; gBOLD, global blood‐oxygen‐level‐dependent signals; HAMA, hamilton anxiety rating scale; HAMD, hamilton depression rating scale; IP, inactive patients; L, left; PSQI, pittsburgh sleep quality index; QoL AP, quality of life questionnaire appearance; QoL VF, quality of life questionnaire visual function; R, right. A significant result was represented by an asterisk (*), and the absence of this symbol indicates a non‐significant result. *p < 0.05, **p < 0.01, ***p < 0.001.

GS function analysis revealed correlations with multiple clinical indicators of the participants (Figures 3, 4, 5). In the AP group, ALPS_L, ALPS_R, and the ALPS index were positively correlated with GO‐QoL VF (*R* = 0.561, *p* = 0.001; *R* = 0.500, *p* = 0.007; *R* = 0.592, *p* = 0.001). However, ALPS_L and ALPS were significantly negatively correlated with HAMA (*R* = −0.319, *p* = 0.010; *R* = −0.297, *p* = 0.016) (Figures 4 and 5A).

Additionally, higher HAMD and HAMA scores in the IP group were also significantly positively correlated with higher sleep scores (*R* = 0.664, *p* = 0.001; *R* = 0.657, *p* = 0.001) (Figures 3 and 5B). Similar patterns were observed in the AP group (*R* = 0.762, *p* < 0.001; *R* = 0.787, *p* < 0.001). These results suggest a potential mechanistic relationship between glymphatic function and clinical manifestations in TAO patients.

### 3.6 Relationship Between GS Indices

**FIGURE 6:**
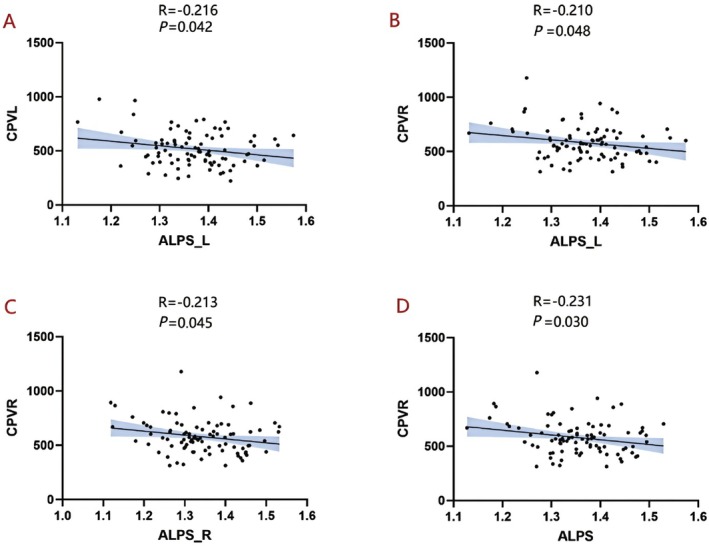
Relationships between CPV and ALPS. (A) In TAO patients, CPV_L was negatively correlated with ALPS_L (R = –0.216, *p* = 0.042). (B–D) CPV_R was negatively correlated with three ALPS indices (R = –0.210, *p* = 0.048; R = –0.213, *p* = 0.045; R = –0.231, *p* = 0.030). ALPS, along the perivascular space; CPV, choroid plexus volume; L, left; R, right.

In patients with TAO, a negative correlation was observed between the CPV_R and the ALPS index—both ALPS_L, ALPS_R, and the whole‐brain ALPS (*R* = −0.210, *p* = 0.048; *R* = −0.213, *p* = 0.045; *R* = −0.231, *p* = 0.030) (Figure 6).

## 4 Discussion

This study represents a novel attempt to systematically reveal the functional abnormalities of the central GS in patients with TAO by using three non‐invasive multimodal MRI techniques. Our research observed significant changes in GS dysfunction markers in both the IP and AP patient groups. Specifically, compared to matched HC, both AP and IP patients exhibited a significant reduction in the ALPS index. Both patient groups also exhibited lower gBOLD‐CSF values. Our preliminary findings provide relevant evidence for the potential neurobiological basis of TAO.

The observed changes in these indices in TAO patients suggest impaired GS function, including disrupted CSF production and glymphatic flow. This represents a novel neurobiological mechanism that could underpin the symptoms of TAO. The GS is essential for removing metabolic waste and preserving homeostasis in the CNS. Therefore, damage to this system could lead to the accumulation of neurotoxic substances, creating a vicious cycle of GS dysfunction and damage.

Although there was no statistically significant difference in the ALPS index between the AP and IP groups, both were significantly lower than the HC. This could be linked to the fact that GS dysfunction persists across different stages of TAO, including both the active and inactive phases. We speculate that the mechanisms underlying GS damage may differ across these stages. In the active phase, inflammatory cytokines cause edema and structural disruption of the perivascular space (PVS) walls [34, 35]. In the inactive phase, collagen deposition and basement membrane thickening lead to reduced elasticity and PVS stiffening [36]. This structural alteration may persistently impair the efficiency of CSF and interstitial fluid exchange, creating a pathological “second hit” model. Crucially, both outcomes—whether through altered permeability or reduced compliance—physically hinder the pulsatile flow of cerebrospinal fluid within the PVS, which is the primary driver of glymphatic exchange measured by the ALPS index. Thus, despite different initial biology, both mechanisms disrupt fluid dynamics in a manner that manifests as a similar quantitative reduction in the ALPS index. These observations suggest that monitoring GS function could serve as a novel biomarker for assessing the degree of neurodamage in TAO.

In this study, we observed that the ALPS index in the left hemisphere was higher than that in the right hemisphere in both the AP and IP groups. This interhemispheric difference is consistent with previous findings in the literature [37]. Given that all participants were right‐handed, this asymmetry may be attributed to right‐handedness and associated hemispheric dominance. In right‐handed individuals, the left hemisphere is typically dominant for language and fine motor control [38]. Such functional specialization may be underpinned by thicker or more coherent fiber bundles that are essential for efficient GS function [39], thereby yielding elevated ALPS indices.

Although the CPV_R in both the AP and IP groups showed an increasing trend, the difference did not reach statistical significance. Preclinical studies have characterized the CP as a key mediator connecting peripheral and central inflammation [40]. It plays a crucial role in maintaining brain homeostasis and constitutes a major interface between the peripheral and CNS [41, 42]. The trend towards CPV enlargement in our TAO patients could hypothetically be linked to the systemic inflammatory state characteristic of this autoimmune disease. This suggests that peripherally produced inflammatory factors may induce adaptive or pathological changes in the CP [43]. In chronic inflammatory conditions, CP epithelial cells may undergo compensatory hypertrophy to cope with impaired CSF secretion. These changes are aimed at preserving brain homeostasis by enhancing cerebrospinal fluid secretion or altering its composition to facilitate the clearance of inflammatory mediators. Further research is warranted to elucidate the role of the CP in TAO and its potential interaction with the GS clearance pathway.

Notably, this study revealed a significant negative correlation between enlarged CPV and reduced ALPS index in patients with TAO, consistent with prior research findings [44, 45]. Although the absolute |*R*| < 0.3 indicates a weak linear relationship, this statistical significance still suggests a potential link between altered cerebrovascular parameters and GS dysfunction. These results imply that changes in CSF production and drainage dynamics may modulate GS activity, possibly contributing to the pathophysiology of TAO. However, further studies with larger samples or complementary imaging modalities are needed to validate and extend these findings.

This study also suggests a potential network linking neuropsychiatric symptoms and GS dysfunction in TAO patients. We found strong correlations between HAMD/HAMA scores and sleep scores, indicating that the psychological symptoms in TAO patients may be influenced by a combination of neuroinflammation; pro‐inflammatory cytokines can affect the hypothalamic–pituitary–adrenal axis and the limbic system, increasing the risk of depression and anxiety [46, 47, 48], while also exacerbating disruptions in the sleep–wake cycle [49]. Furthermore, evidence suggests a pathogenic bidirectional relationship between sleep disturbances and GS dysfunction. Given the system's maximal activity during slow‐wave sleep, TAO‐induced sleep abnormalities may substantially impair GS clearance [50]. Conversely, GS dysfunction may interfere with sleep‐regulating homeostatic mechanisms, creating a self‐perpetuating pathological loop that progressively worsens both conditions [50]. This bidirectional mechanism may serve as a key explanation for the reduced quality of life in TAO patients.

This study recognizes a few key limitations. First, the cross‐sectional design limits any causal inferences from the observed associations. Longitudinal studies that track patients from the active to the inactive phase are needed to better understand the temporal progression and directionality of these relationships, thereby clarifying the nature of the observed changes. Second, the relatively limited sample size may restrict the broader applicability of the study's findings and preclude a meaningful analysis of potential subgroups (e.g., based on the severity of neuropsychiatric symptoms). Future investigations should employ larger, more diverse cohorts to validate these preliminary findings, strengthen the generalizability of the observed relationships, and enable such subgroup analyses. Finally, the GS assessment in this study remains indirect. Although ALPS, CPV and gBOLD‐CSF coupling represent the currently recognized non‐invasive proxies, they do not constitute a direct quantification of glymphatic fluid movement. Future work that can couple these imaging markers with intrathecal contrast‐based or other invasive gold‐standard techniques is warranted to validate their mechanistic specificity.

In conclusion, this study provides a multi‐parameter approach using multimodal non‐invasive imaging proxies to comprehensively assess GS function in TAO patients. These findings contribute to a deeper understanding of the pathophysiological processes of TAO and promote the development of integrated therapeutic strategies.

## Author Contributions

L.Z.: conceptualization, data curation, formal analysis, methodology, writing‐original draft. J.Y.: data curation, formal analysis, methodology, visualization. Y.Q.: conceptualization, formal analysis, validation. Y.L.: resources, conceptualization. J.L.: funding acquisition, conceptualization, supervision. All authors reviewed the manuscript.

## Disclosure

The authors have nothing to report.

## Ethics Statement

This study was approved by the Ethics Review Committee of the Second Xiangya Hospital of Central South University (reference number: LYEC2025‐0027).

## Conflicts of Interest

The authors declare no conflicts of interest.

## Supporting information


**Figure S1:** cns70650‐sup‐0001‐FigureS1.tif.

**Figure S2:** cns70650‐sup‐0002‐FigureS2.tif.

**Table S1:** cns70650‐sup‐0003‐TableS1.docx.

**Table S2:** cns70650‐sup‐0004‐TableS2.docx.

## Data Availability

The data that support the findings of this study are available on request from the corresponding author. The data are not publicly available due to privacy or ethical restrictions.
