# Supplementary figures and images for: Glymphatic System Dysfunction in Thyroid‐Associated Ophthalmopathy: A Multimodal MRI Study
Source: CNS Neurosci Ther. 2025 Nov 9;31(11):e70650. doi: 10.1111/cns.70650 (PMC12597980; doi:10.1111/cns.70650)

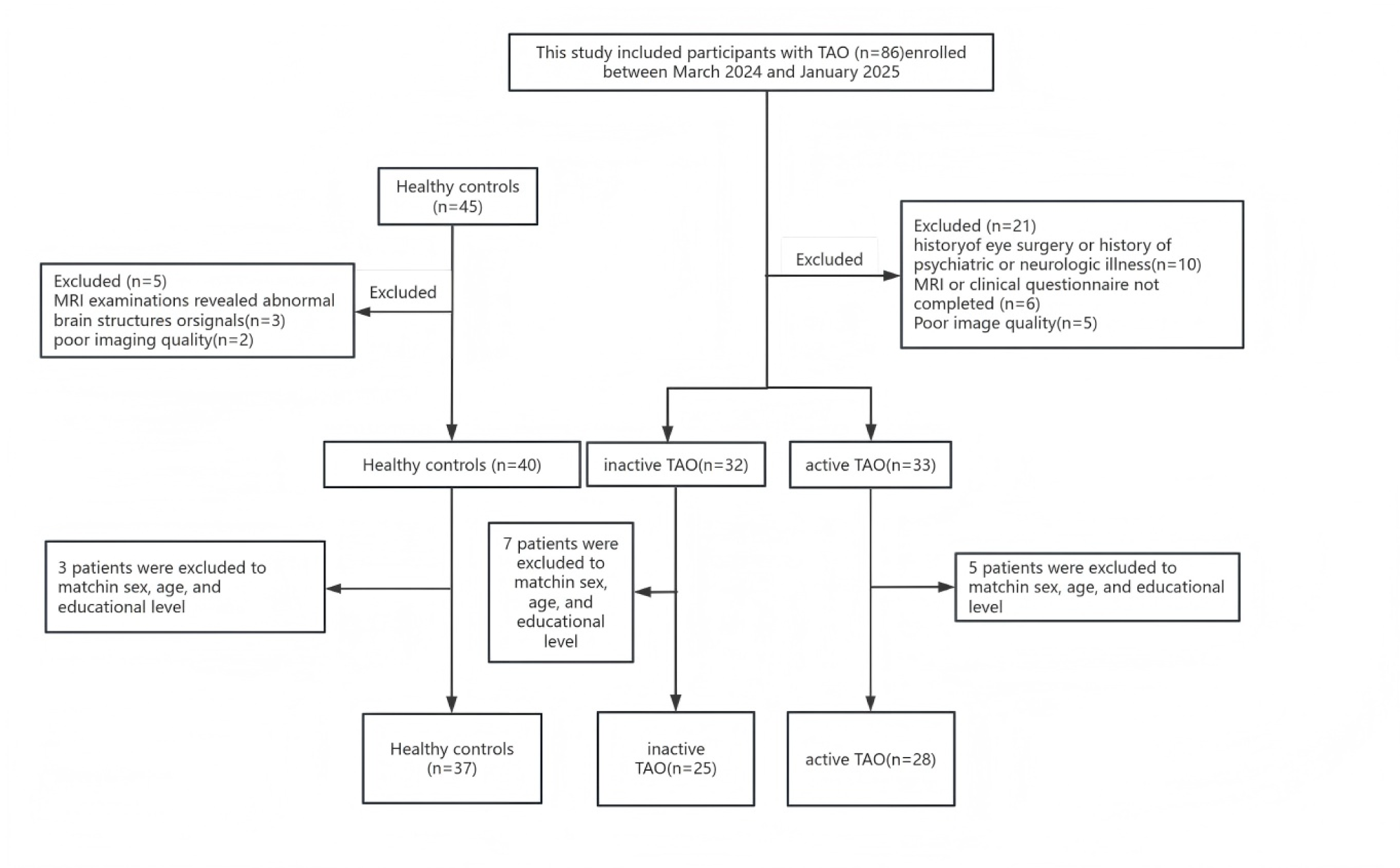

Supplement: Supplementary file 1 — Figure S1: cns70650‐sup‐0001‐FigureS1.tif. [file CNS-31-e70650-s003.tif]

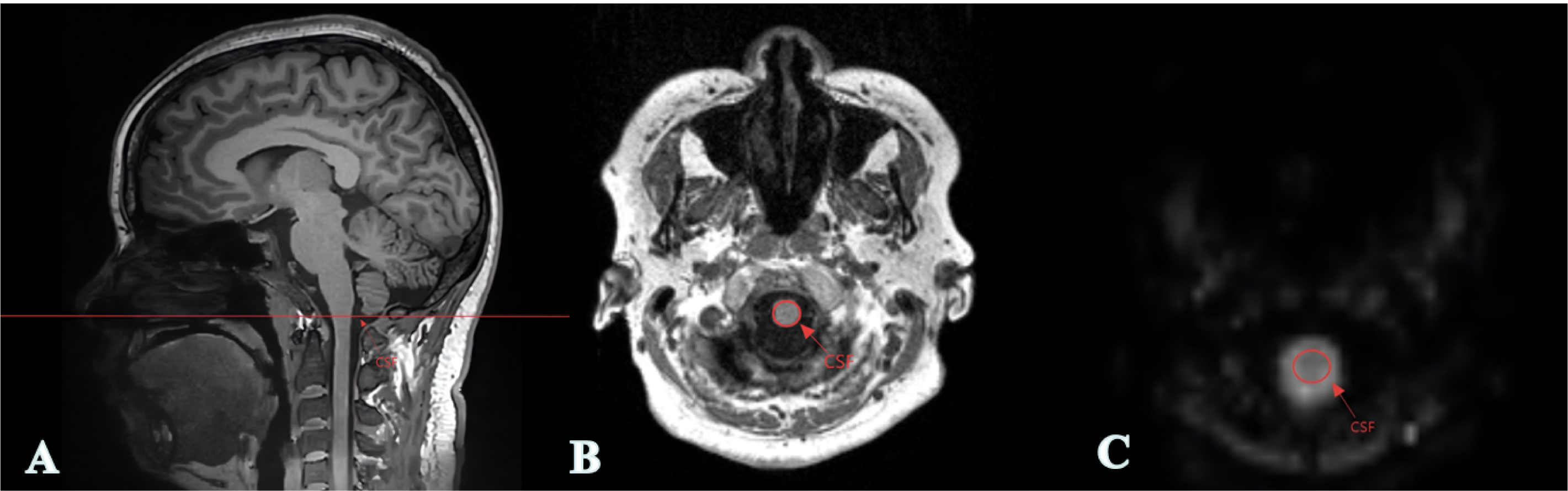

Supplement: Supplementary file 2 — Figure S2: cns70650‐sup‐0002‐FigureS2.tif. [file CNS-31-e70650-s002.tif]
