# Supplementary material for: Glymphatic System Dysfunction in Thyroid‐Associated Ophthalmopathy: A Multimodal MRI Study
Source: CNS Neurosci Ther. 2025 Nov 9;31(11):e70650. doi: 10.1111/cns.70650 (PMC12597980; doi:10.1111/cns.70650)
Supplement: Supplementary file 3 — Table S1: cns70650‐sup‐0003‐TableS1.docx. [file CNS-31-e70650-s004.docx]

Table S1. Multimodal magnetic resonance scanning parameters.

|  | TR  (ms) | TE  (ms) | number of slices | slice thickness (mm) | FOV  （mm） | acquisition matrix | flip angle (°) | number of diffusion gradient directions | b value  (s/mm^2^) |
| --- | --- | --- | --- | --- | --- | --- | --- | --- | --- |
| T2WI | 5277 | 121 | 22 | 5.5 | 230X200 | 334×384 | 90 | / | / |
| T1WI | 7.9 | 3.1 | 180 | 1 | 256×230 | 230×256 | 10 | / | / |
| fMRI | 2346 | 30 | 36 | 4 | 192×192 | 96x96 | 80 | / | / |
| DTI | 5067 | 71 | 45 | 3 | 204x204 | 68x68 | 90 | 64 | 0, 1000 |

Abbreviations: FOV, field-of-view; TE, echo time; TR, repetition time; DTI, diffusion tensor imaging; fMRI, functional magnetic resonance imaging.
