# Supplementary material for: Glymphatic System Dysfunction in Thyroid‐Associated Ophthalmopathy: A Multimodal MRI Study
Source: CNS Neurosci Ther. 2025 Nov 9;31(11):e70650. doi: 10.1111/cns.70650 (PMC12597980; doi:10.1111/cns.70650)
Supplement: Supplementary file 4 — Table S2: cns70650‐sup‐0004‐TableS2.docx. [file CNS-31-e70650-s001.docx]

Table S2. Demographic and clinical characteristics of TAO patients and HC.

| Characteristics | AP | IP | HC | *P* value | |
| --- | --- | --- | --- | --- | --- |
|  |  |  |  | AP vs IP | AP vs IP vs HC |
| Sex, Male (n, %) | 7 (25%) | 5 (20%) | 10 (27%) | 0.671 | 0.760 |
| Age (year) | 38(28; 52) | 29(25; 42) | 33(26; 52) | 0.165 | 0.417 |
| Disease duration (months) | 8(4; 14) | 11(6; 24) | / | 0.212 | / |
| educational attainment (year) | 14(9; 16) | 16(11; 16) | 16(10; 16) | 0.357 | 0.598 |
| TSH (mIU/L) | 0.24(1.04; 1.43) | 0.09(0.01; 1.18) | / | 0.316 | / |
| fT3 (pmol/L) | 4.31(2.36; 5.36) | 3.82(2.23; 5.31) | / | 0.756 | / |
| fT4 (pmol/L) | 4.49(2.09; 13.15) | 3.40(1.61; 9.58) | / | 0.689 | / |
| TRAb (IU/L) | 12.60(3.70; 23.04) | 11.58(2.46; 21.10) | / | 0.296 | / |
| CAS | 4(3; 5) | 2(1; 2) | / | < 0.001 | / |
| QoL VF | 21(18; 22) | 22(22; 22) | / | 0.003 | / |
| QoL AP | 17(12; 20) | 18(13; 20) | / | 0.629 | / |
| HAMD | 9(7; 11) | 9(6; 11) | 3(2; 4) | 0.938 | < 0.001 |
| HDMA | 12(7; 15) | 9(6; 15) | 3(2; 4) | 0.408 | < 0.001 |
| PSQI | 9(6; 11) | 8(6; 10) | 4(3; 6) | 0.65 | < 0.001 |

Abbreviations: AP, active patients; CAS, clinical activity score; HAMD, hamilton depression rating scale; HAMA, hamilton anxiety rating scale; HC, healthy controls; IP, inactive patients; PSQI, pittsburgh sleep quality index; QoL AP, quality of life questionnaire appearance; QoL VF, quality of life questionnaire visual function; TSH, thyroid-stimulating hormone; fT3, free triiodothyronine; fT4, free thyroxine; TRAb; Thyroid-Stimulating Hormone Receptor Antibodies.
